# Male Mice Express Spermatogenic Cell-Specific Triosephosphate Isomerase Isozymes

**DOI:** 10.1002/mrd.22217

**Published:** 2013-08-19

**Authors:** Takashi W Ijiri, Melissa L Vadnais, Angel M Lin, Andy P Huang, Wenlei Cao, Tanya Merdiushev, George L Gerton

**Affiliations:** 1 Center for Research on Reproduction and Women's Health Perelman School of Medicine, University of PennsylvaniaPhiladelphia, Pennsylvania; 2 Department of Molecular Biosciences, Kyoto Sangyo UniversityKyoto, Japan; 3 Department of Obstetrics and Gynecology Perelman School of Medicine, University of PennsylvaniaPhiladelphia, Pennsylvania

**Keywords:** sperm, fibrous sheath, flagellum, triosephosphate isomerase, spermatogenesis

## Abstract

Triosephosphate isomerase 1 (TPI1) is a member of the glycolytic pathway, which is a critical source of energy for motility in mouse sperm. By immunoblotting, we detected two male, germ line-specific TPI1 bands (M_r_ 33,400 and 30,800) as well as the somatic-type band (M_r_ 27,700). Although all three bands were observed in spermatogenic cells, somatic-type TPI1 disappeared from sperm during epididymal maturation. In vitro dephosphorylation analysis suggested that the two male, germ line-specific TPI1 bands were not the result of phosphorylation of the 27,700 M_r_ TPI1 band. The M_r_ 33,400; 30,800; and 27,700 TPI1 bands corresponded to the respective sizes of the proteins predicted to use the first, second, and third possible initiation codons of the *Tpi1* cDNA. We performed immunofluorescence on epididymal sperm and determined that TPI1 specifically localized in the principal piece. The antibody staining was stronger in cauda epididymal sperm than in caput epididymal sperm, a finding consistent with the identification of TPI1 as a cauda epididymal sperm-enriched protein. Immunofluorescence with sodium dodecyl sulfate (SDS)-insoluble flagellar accessory structures showed a strong TPI1 signal only in the principal piece, indicating that TPI1 is a component of the fibrous sheath. Northern blot hybridization detected longer *Tpi1* transcripts (1.56 kb) in mouse testis, whereas somatic tissues had shorter transcripts (1.32 kb). As there is only one triosephosphate isomerase gene in the mouse genome, we conclude that the three variants we see in sperm result from the use of alternative translation start codons in spermatogenic cells. *Mol. Reprod. Dev. 80: 862–870, 2013. © 2013 The Authors*. Published by Wiley Periodicals, Inc. This is an open access article under the terms of the Creative Commons Attribution-NonCommercial-NoDerivs License, which permits use and distribution in any medium, provided the original work is properly cited, the use is non-commercial and no modifications or adaptations are made.

## INTRODUCTION

Male germ cells have to produce ATP from available substrates for their cellular activity and for differentiation into spermatozoa. Although spermatocytes and spermatids prefer oxidative phosphorylation as a means for ATP production (Nakamura et al., [Bibr b24],[Bibr b25]), studies have suggested that sperm of many mammals switch to glycolysis for ATP production during epididymal maturation (Storey and Kayne, 1975). During spermatogenesis, a number of somatic-type glycolytic enzymes are replaced by male germ cell-specific isozymes, many of which are found in association with the fibrous sheath of the flagellar principal piece (McCarrey and Thomas, 1987; Sakai et al., 1987; Welch et al., 1992a; Mori et al., 1993).

Glycolysis clearly plays an essential role as an energy pathway to fuel basal motility in mouse sperm, as is evident by studies of male mice with genetic deletions of the sperm-specific forms of key glycolytic enzymes, such as glyceraldehyde 3-phosphate (GAP) dehydrogenase-S, lactate dehydrogenase C, and phosphoglycerate kinase-2 (Miki et al., 2004; Odet et al., 2008; Danshina et al., 2010). When oxidative phosphorylation is suppressed by carbonyl cyanide m-chlorophenylhydrazone, sperm motility and ATP production are not affected; on the other hand, sperm motility and ATP content are negatively impacted when glucose is replaced in the medium by a non-hydrolyzable analog, 2-deoxyglucose, even though pyruvate or lactate is also present to fuel mitochondrial respiration (Mukai and Okuno, 2004).

Glycolysis converts glucose into pyruvate via a series of reactions by ten enzymes, yielding two ATPs from a single glucose molecule. During glycolysis, aldolase converts fructose 1,6-diphosphate to GAP and dihydroxyacetone phosphate (DHAP). Only GAP is a direct substrate for GAP dehydrogenase, thus triosephosphate isomerase 1 (TPI1) is required to convert DHAP to GAP. The conversion reaction is highly efficient, and is also very reversible, forming DHAP from GAP in cells involved in gluconeogenesis. TPI1 has been found in most organisms, and has been studied in the human and rat male germ lines (Russell and Kim, 1996; Auer et al., 2004); yet, little is known about the mouse enzyme. We previously reported that mouse TPI1 was present in the sperm flagellar accessory structures and was also identified as a cauda epididymal sperm-enriched protein (Cao et al., 2006; Ijiri et al., 2011). The glycolytic enzyme aldolase has male germ line-specific isozymes (ALDOA, ALDOAV2, ALDOART1, ALDOART2), and anti-muscle aldolase or anti-ALDOA antibodies detected the ubiquitous shorter (M_r_ 39,000) and longer (M_r_ 50,000) male germ cell-specific isozymes (Vemuganti et al., 2007; Arcelay et al., 2008; Ijiri et al., 2011). Similar to aldolase, immunoblotting showed two male germ line-specific M_r_ 33,400 and 30,800 TPI1 bands as well as the somatic-type M_r_ 27,700 band (Ijiri et al., 2011).

In this study, we investigated mouse TPI1 further by analyzing protein distribution in somatic and reproductive tissues. We also examined the localization of this protein during epididymal maturation using caput and cauda epididymal sperm. As it is reported that multiple glycolytic enzymes are tightly bound to the fibrous sheath in mouse (Krisfalusi et al., 2006), we also performed immunofluorescence with sodium dodecyl sulfate (SDS)-insoluble flagellar accessory structures to determine if TPI1 was specifically localized in the fibrous sheath. We demonstrated that in the mouse, TPI1 existed as three isozymes (two male germ line-specific and one somatic tissue type). The male germ line-specific isozymes were present in spermatogenic cells and epididymal sperm, and associate with the caput and cauda epididymal sperm flagellum. Furthermore, the mouse *Tpi1* gene sequence and transcripts were examined by Northern blot hybridization analyses and Rapid Amplification of cDNA Ends (RACE), respectively, to elucidate how TPI1 isozymes were produced in male mouse germ cells. We present evidence that the three variants present in sperm result from the use in spermatogenic cells of alternative translation start codons for *Tpi1*, the only triosephosphate isomerase gene in the mouse genome.

## RESULTS

### The Male Mouse Germ Line Exhibits Two Specific TPI1 Isozymes

**Figure 1:**
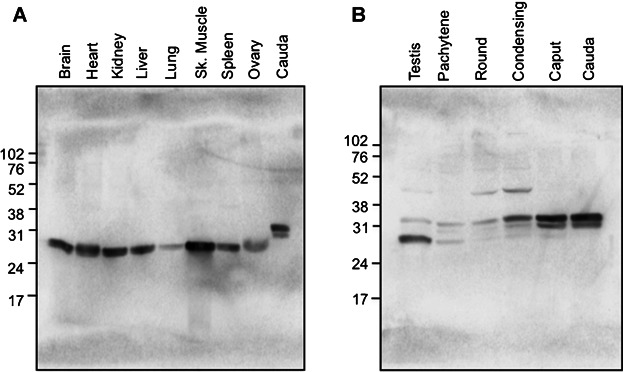
SDS–PAGE and immunoblotting of protein extracts from somatic tissues, reproductive tissues, and spermatogenic cells. Immunoblotting with protein extracts from brain, heart, kidney, liver, lung, skeletal muscle, spleen, ovary, and cauda epididymal sperm (A). Immunoblotting with protein extracts from testis, pachytene spermatocytes, round spermatids, condensing spermatids, caput epididymal sperm, and cauda epididymal sperm (B). The somatic-type TPI1 protein (M_r_ 27,700) was detected in all protein samples examined, except cauda epididymal sperm, using an anti-TPI1 antibody. In spermatogenic cells and sperm, two male germ line-specific TPI1 bands (M_r_ 33,400 and 30,800) were present.

To determine where TPI1 was expressed and whether or not a specific isozyme(s) existed, we performed SDS–polyacrylamide gel electrophoresis (SDS–PAGE) and immunoblotting with various somatic and reproductive tissues. The somatic-type TPI1 protein (M_r_ 27,700) was detected in somatic tissues, ovary, and testis (Fig. 1). In spermatogenic cells and sperm, we found two male germ line-specific TPI1 bands (M_r_ 33,400 and 30,800). Although all three bands were observed in germ cells from pachytene spermatocytes to caput epididymal sperm, somatic-type TPI1 disappeared from sperm during epididymal maturation. The two male germ line-specific bands increased gradually in relative amounts, becoming most abundant in cauda epididymal sperm.

### The Male Germ Line-Specific TPI1 Isozymes Do Not Result From Differential Phosphorylation

**Figure 2:**
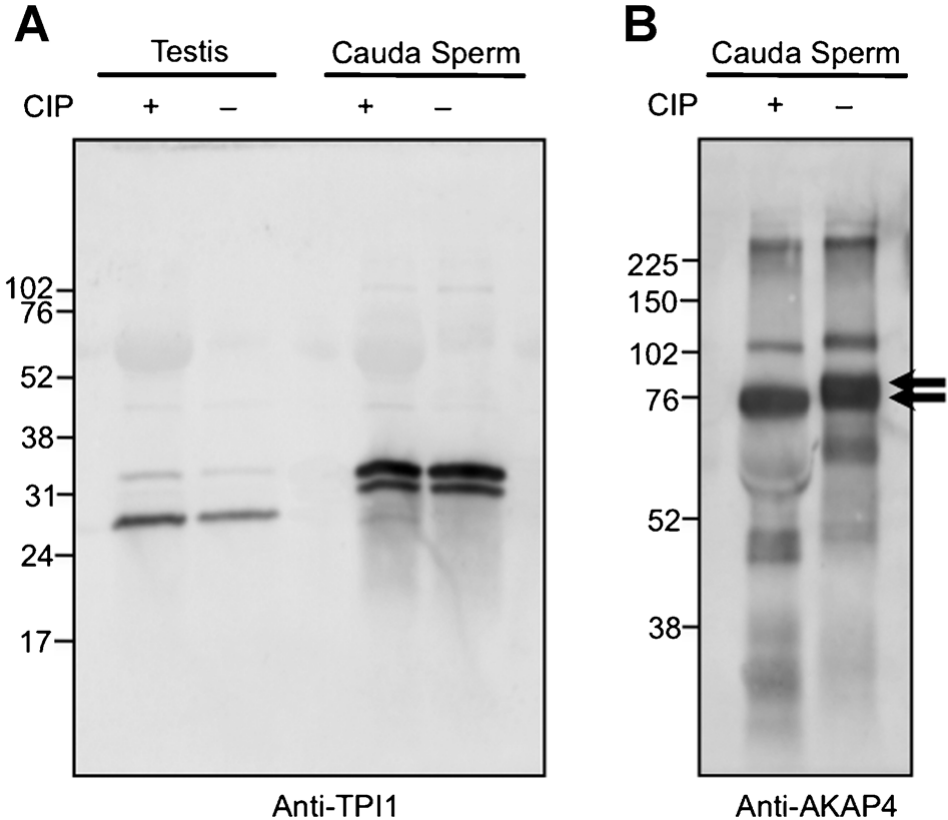
In vitro dephosphorylation of TPI1 in mouse sperm. CIP treatment of testis and cauda epididymal sperm proteins probed with anti-TPI1 antibody (A) and cauda epididymal sperm proteins probed with anti-AKAP4 antibody as a positive control (B). The two arrows point to the two AKAP4 forms present prior to dephosphorylation. In vitro dephosphorylation analysis indicated that the two male germ line-specific TPI1 isozymes did not result from differences in phosphorylation.

Calf intestinal alkaline phosphatase (CIP) was used to determine if the differences in relative mobility of the three TPI1 isozymes were due to differential phosphorylation. Proteins extracted from testis and mature epididymal sperm were treated with alkaline phosphatase and then examined by immunoblotting with anti-TPI1 antibody. As a positive control, cauda epididymal sperm proteins were probed with anti-AKAP4 antibody with and without phosphatease treatment to demonstrate a shift in mobility caused by dephosphorylation (Johnson et al., 1997). In vitro dephosphorylation analysis did not produce any change in the TPI1 relative mobility, indicating that the two male germ line-specific TPI1 isozymes did not result from differences in phosphorylation status (Fig. 2).

### Northern Blot Hybridization Detects Longer Tpi1 Transcripts in the Mouse Testis

**Figure 3:**
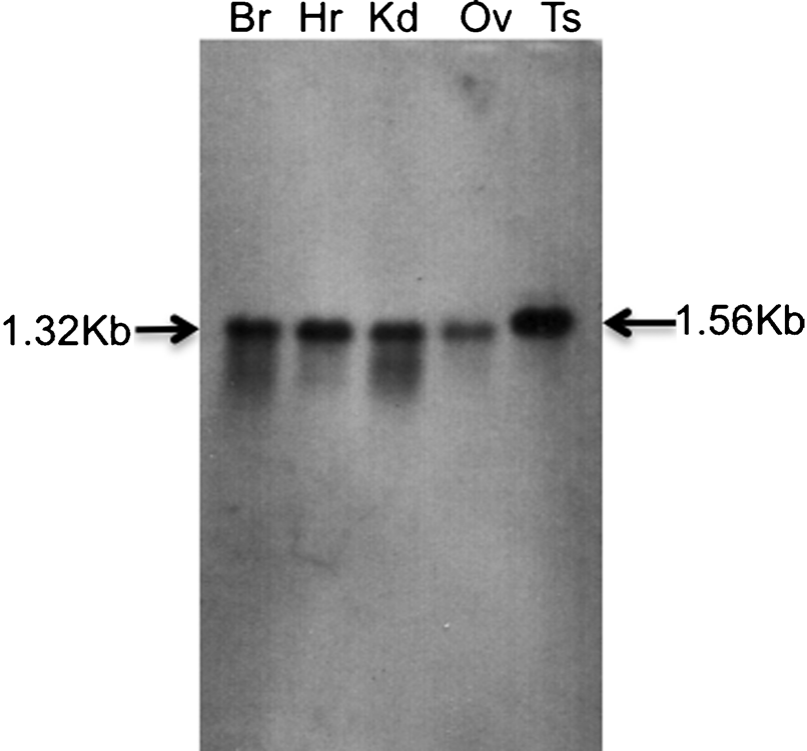
Northern blot hybridization with a *Tpi1* antisense probe. Northern blot hybridization was performed to confirm how many male germ line-specific *Tpi1* bands were present. Message RNA from brain (Br), heart (Hr), kidney (Kd), ovary (Ov), and testis (Ts) were examined. This method detected a longer, testis-specific *Tpi1* transcript of 1.56 kb; somatic tissues and ovary contained only a 1.32-kb transcript.

To determine if the existence of male germ line-specific *Tpi1* transcripts could explain the differences seen in the proteins extracted from spermatogenic cells, we performed Northern blot hybridization on messenger RNA from brain, heart, kidney, ovary, and testis. A single band at 1.32 kb was noted in brain, heart, kidney, and ovary (Fig. 3). In the testis, a larger but broader single band was also observed. The center of this band corresponded to a size of 1.56 kb.

### The Male Mouse Germ Line Expresses a Spermatogenic-Specific Tpi1 mRNA Variant

Recognizing that the larger-sized mRNAs in the testis could result from extension at either end of the message, cDNAs were created and 5′ RACE was performed to identify potential initiation codons. Two database cDNA sequences exist for mouse *Tpi1*. The original sequence, NM_009415.1, was based upon predictions from the genomic sequence on mouse chromosome 6 and has subsequently been replaced by an updated, predicted sequence, NM_009415.2, which lacks the 5′-most in-frame, upstream stop codon that is present in NM_009415.1. Using 5′ RACE, we found two different transcripts in mouse spermatogenic cell cDNA. Transcript 1 contained all three potential initiation codons and an in-frame, upstream stop codon, whereas, transcript 2, like NM_009415.2, lacked the in-frame, upstream stop codon and the first potential initiation codon (Suppl. Fig. S1).

In rat, testicular *Tpi1* mRNA contains an additional 100 bases in the 3′-untranslated region (UTR) that is specific to male germ cells (Russell and Kim, 1996). We tested if mouse *Tpi1* mRNA contained a region similar to the 3′ UTR of rat *Tpi1* using 3′ RACE. The predicted mouse *Tpi1* sequence, NM_009415.2, includes the 3′-UTR extension that is homologous to the germ line-specific 3′-UTR of rat *Tpi1* cDNA; however, our 3′-RACE analyses did not detect the extended sequence (Suppl. Fig. S2). Sequences from ten different 3′-RACE clones terminated at the same point predicted by NM_009415.1, indicating that unlike the rat, the mouse does not express a male germ line-specific 3′-UTR. The nucleotide sequences did contain a canonical polyadenylation signal for *Tpi1* (Suppl Fig. S2, box).

### TPI1 Isozymes Likely Result From Three Different Initiation Codons

To determine the basis for the differences in M_r_ of the germ-cell forms of TPI1, open reading frames from the full-length mouse *Tpi1* cDNA were predicted based on our 5′-RACE results and NM_009415.1. Three potential initiation codons were identified in the *Tpi1* cDNA. The M_r_ 33,400; 30,800; and 27,700 TPI1 bands corresponded to the sizes of the proteins predicted to use the first (299 amino acids long, calculated molecular weight: 32,192), second (286 amino acids, calculated molecular weight: 30,890), and third (249 amino acids, calculated molecular weight: 26,712) initiation codons of the *Tpi1* cDNA, respectively (Suppl. Fig. S3; the two orange boxes indicate the putative translation initiation codons of the two male germ line-specific TPI1 isozymes, and the green box is the known translation initiation codon of somatic-type TPI1). An in-frame stop codon is located 42 bp upstream from the first putative start codon at nucleotide 141 (red box). The second and third initiation codons contain a strong Kozak consensus sequence following the (gcc)gccRccAUGG sequence, consisting of a purine in position −3 and a guanine in position +4 (Kozak, 1987). The first initiation codon has a weak Kozak consensus sequence, and does not contain a purine in position −3.

### Germ Line-Specific TPI1 Isozymes Partition to the Flagellar Accessory Structures

**Figure 4:**
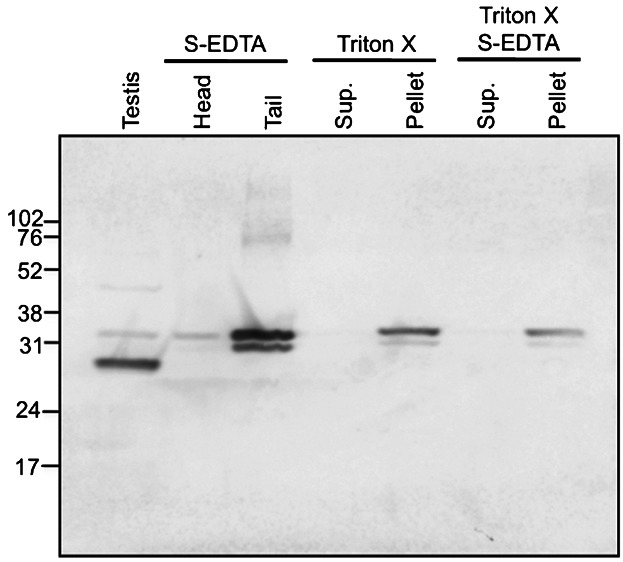
SDS–PAGE and immunoblotting of detergent-extracted sperm proteins. Immunoblotting on SDS-insoluble head and tail proteins, Triton X-100-soluble and -insoluble, whole sperm proteins, and Triton X-100/S-EDTA-soluble and -insoluble whole sperm proteins was performed. The two male germ line-specific bands (M_r_ 33,400 and 30,800) were enriched in the S-EDTA-treated sperm tail fraction, in Triton X-100-insoluble sperm proteins, and in Triton X-100/S-EDTA-insoluble sperm proteins.

To assess if TPI1 associated with components of the flagellar accessory structures such as the fibrous sheath, cauda epididymal sperm proteins were fractionated by extracting with S-EDTA (1% SDS, 75 mM NaCl, 10 mM EDTA, pH 6.0), 0.1% Triton X-100, or 0.1% Triton X-100 followed by S-EDTA; resulting fractions were analyzed by immunoblotting with anti-TPI1 antibody. S-EDTA solubilizes these components and dissolves the axoneme, leaving behind flagellar accessory structures such as the fibrous sheath, outer dense fibers, and remnants of the mitochondrial sheath. Triton X-100, on the other hand, solubilizes membranes and releases soluble, cytoplasmic components but leaves the axoneme intact. The two male germ line-specific bands of M_r_ 33,400 and 30,800 were enriched in the S-EDTA-treated, sperm-tail fraction (Fig. 4). The upper TPI1 band (M_r_ 33,400) was faintly observed in the sperm head fraction. The two germ line-specific isozyme bands were detected in Triton X-100/S-EDTA insoluble sperm proteins, but not in the soluble fraction.

### TPI1 Localizes to the Principal Piece of Mouse Epididymal Sperm

**Figure 5:**
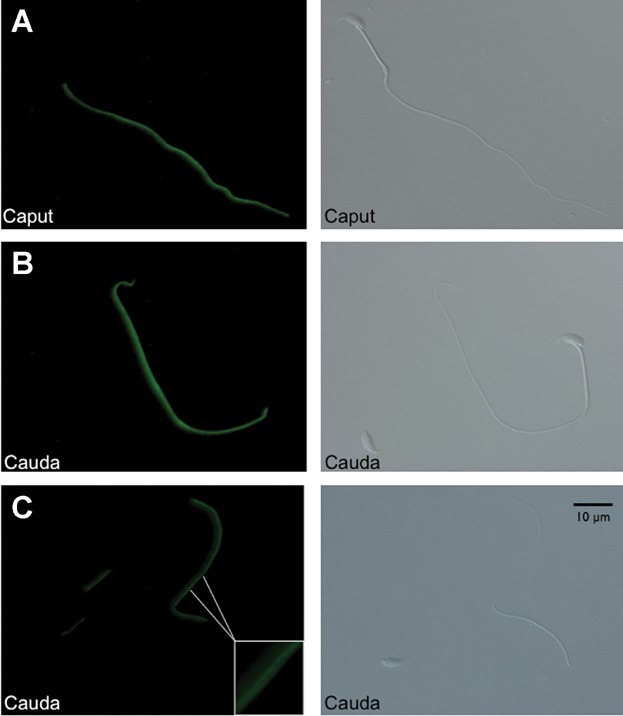
Indirect immunofluorescence with anti-TPI1 antibody. TPI1 was detected in the principal piece of caput epididymal sperm (A), cauda epididymal sperm (B), and SDS-insoluble tail accessory structures of cauda epididymal sperm (C). Scale bar, 10 µm.

Indirect immunofluorescence was performed on caput and cauda epididymal sperm treated with anti-TPI1 antibody (Fig. 5), then exposed to fluorescence (left panels) and Nomarski differential interference contrast (right panels) microscopy. TPI1 specifically localized to the principal piece of caput epididymal sperm (Fig. 5A) and cauda epididymal sperm (Fig. 5B). After solubilization of sperm with S-EDTA, TPI1 remained detectable in the principal piece of the SDS-insoluble flagellar accessory structures (Fig. 5C). Fluorescence appeared to be associated with the longitudinal columns of the fibrous sheath. No signal was observed in normal-IgG controls (data not shown).

## DISCUSSION

Glycolysis plays an important role in energy production with mammalian sperm. All glycolytic enzymes play key roles, and the requirement for TPI1 in the male germ cell is evident from inhibitor studies, which demonstrated that a TPI1 inhibitor decreases rat sperm motility by 50%, consistent with the hypothesis that the conversion of DHAP to GAP increases the efficiency of the glycolytic process (Bone and Cooper, 2000; Bone et al., 2001). Although some information is available concerning TPI1 in sperm from a limited number of species, an extensive analysis of the isoforms present and the localization of the protein in sperm have not been published. We previously reported that mouse TPI1 was present in the sperm flagellar accessory structures and was identified as a cauda epididymal sperm-differential protein by proteomic analysis (Cao et al., 2006; Ijiri et al., 2011). Here, we have shown that TPI1 is restricted to the principal piece of the sperm flagellum, and is tethered to the fibrous sheath.

Somatic TPI1 was present in pachytene spermatocytes, but declined in amount as spermatogenic cells matured. Conversely, late-meiotic and post-meiotic male germ cells expressed two specific TPI1 isozymes (M_r_ 33,400 and 30,800; Fig. 1). During epididymal maturation, the two male germ line-specific TPI1 isozymes were enriched while the somatic-type disappeared. Cauda epididymal sperm exhibited only the two male germ line-specific TPI1s, suggesting that these isozymes are important for sperm function. Loss of the somatic form during spermatogenesis and epididymal maturation probably reflects remodeling of the male germ cell through the shedding of excess cytoplasm in residual bodies and cytoplasmic droplets from spermatids and cauda epididymal sperm, respectively.

From our studies, we conclude that the M_r_ 33,400; 30,800; and 27,700 TPI1 isozymes corresponded to the sizes of the proteins predicted to use the respective first, second, and third possible initiation codons of the *Tpi1* cDNA (Suppl. Fig. S3). The methionine present at the third possible initiation codon is reportedly used to generate somatic TPI1 (Yuan et al., 1979; Cheng et al., 1990). The use of alternative start codons is similar to the gonadotropin-regulated testicular RNA helicase gene, which results from the utilization of optional translation initiation codons in the rat testis (Sheng et al., 2003). Northern blot hybridization for *Tpi1* mRNA detects longer *Tpi1* transcripts in mouse testis compared to somatic tissue or female reproductive tissues (Fig. 3), and is independent. Additionally, the differences in relative mobilities of the germ line-specific TPI1 proteins were not due to differential phosphorylation (Fig. 2). The expression of sperm-specific glycolytic enzymes from the same gene that is used in somatic cells contrasts to the situation with other glycolytic enzymes. In the cases of lactate dehydrogenase and phosphoglycerate kinase, alternate genes are responsible for the sperm-specific isozymes (Vemuganti et al., 2010). These genes usually originate from retrotransposition events. In the case of triosephosphate isomerase, retrotransposed sequences are present in the human, but these sequences do not appear to be expressed in the testis (Vemuganti et al., 2010). At present, the mechanism regulating the use of alternative start codons in male germ-cell TPI1 isozyme translation is unknown.

Amino terminal extensions are common for proteins that associate with the fibrous sheath in a detergent-resistant manner. Examples of this phenomenon include germ cell-specific hexokinase 1 and GAP dehydrogenase (Welch et al., 1992b; Mori et al., 1998; Travis et al., [Bibr b35],[Bibr b36]). The male germ line-specific N-termini of TPI1, which contain additional 50 and 37 amino acids, are not predicted to have an alpha helical structure (data not shown) as do the tethering domains of the regulatory subunits of protein kinase A (Visconti et al., 1997; Miki and Eddy, 1999). To examine this issue further, we determined whether or not these isozymes associated with the fibrous sheath. S-EDTA solubilizes and dissolves the axoneme, leaving behind flagellar accessory structures such as the fibrous sheath, outer dense fibers, and remnants of the mitochondrial sheath. Triton X-100 solubilizes membranes and releases soluble cytoplasmic components, but leaves the axoneme intact. The germ line-specific TPI1 bands (M_r_ 33,400 and 30,800) were detected in the S-EDTA-treated sperm tail fraction and Triton X-100/S-EDTA insoluble sperm proteins, but not in S-EDTA-treated head and Triton X-100/S-EDTA-soluble sperm protein fractions (Fig. 4). Using indirect immunofluorescence with anti-TPI1 antibody, we showed that the enzyme localized to the principal piece of mouse epididymal sperm, and appeared to be present in the longitudinal columns of the fibrous sheath in cauda epididymal sperm (Fig. 5). These results are consistent with studies of other glycolytic enzymes that were found to be tethered to the fibrous sheath of the sperm flagellum (Krisfalusi et al., 2006; Nakamura et al., 2013).

The studies presented here provide evidence for the attachment of another glycolytic enzyme to the fibrous sheath. Together with hexokinase, aldolase, phosphoglycerate kinase, GAP dehydrogenase, enolase, and pyruvate kinase, TPI1 associates with the fibrous sheath to form a specialized “metabolosome” within the sperm flagellum. This compartmentalized and particulate structure forms the primary mechanism for generating ATP within a cell with very little cytoplasm. Additional studies should be performed to define the complete metabolic pathways within the sperm, including the further processing of pyruvate and lactate. Such studies may discover new approaches for contraceptive development. In addition, the sperm flagellum serves as a model for the development of micro- and nanoscale hybrid material systems that can attach biological components, such as enzymes, to inorganic surfaces without compromising catalytic function (Mukai et al., 2013).

## MATERIALS AND METHODS

### Spermatogenic Cell Isolation

All animal procedures were approved by the University of Pennsylvania Institutional Animal Care and Use Committee. Mixed germ cells were prepared from decapsulated testes of adult male mice (Crl:CD1(ICR) retired breeders; Charles River Laboratories, Wilmington, MA) by sequential dissociation with collagenase and trypsin-DNase I (Bellvé et al., 1977). To purify populations of pachytene spermatocytes, round spermatids, and condensing spermatids, mixed germ cells were separated at unit gravity in a 2–4% bovine serum albumin gradient in Eagle's Essential Medium with Earle's Salts (Romrell et al., 1976; Gerton and Millette, 1984). Both the pachytene spermatocyte and round spermatid populations were at least 85% pure, as determined by microscopic examination and differential counting with a hemocytometer; the condensing spermatid population was approximately 40–50% pure, with the balance primarily composed of anucleate residual bodies and round spermatids.

### Purification of Caput and Cauda Epididymal Sperm

Sperm were collected from the caput and caudae epididymides of male mice by cutting the epididymides and extruding the sperm at 37°C into phosphate-buffered saline (PBS; 2.68 mM KCl, 136.09 mM NaCl, 1.47 mM KH_2_PO_4_, 8.07 mM Na_2_HPO_4_, pH 7.4) containing protease inhibitors (Roche Applied Science, Indianapolis, IN). Caput epididymal sperm were purified by centrifugation at 400*g* for 20 min at room temperature through a 35% SupraSperm 100 solution (MidAtlantic Devices, Mt. Laurel, NJ) in PBS. Purified sperm were collected from the pellet, resuspended in PBS at 4°C, counted, and assessed for purity. Cauda epididymal sperm were collected by centrifugation at 800*g* for 5 min at room temperature, resuspended in PBS at 4°C, and counted.

### Purification of SDS-Insoluble Sperm Heads and Tails

Cauda epididymal sperm were homogenized in S-EDTA, layered onto a 1.6 M sucrose cushion in S-EDTA, and centrifuged at 5,000*g* for 1 hr at room temperature (O'Brien and Bellvé, 1980). The SDS-resistant head structures (nuclei) were collected from the pellet. The SDS-resistant tail structures (flagellar accessory structures lacking the plasma membrane and axonemes) were collected from the interface. The purity was visually assessed by light microscopy to determine adequate head and flagellar accessory structure (“tail”) separation.

### Detergent Fractionation of Sperm Cells

Cauda epididymal sperm were collected as described above. One million cells in 100 µl of PBS containing 0.1% Triton X-100 were incubated at room temperature for 10 min, then centrifuged at 10,000*g* for 1 min. Triton X-100-soluble and -insoluble proteins were collected from the supernatant and pellet, respectively. Further detergent extraction was performed on the Triton X-100-insoluble proteins (pellets). One hundred microliters of S-EDTA was added to the Triton X-100-insoluble protein pellet, homogenized, incubated at room temperature for 10 min, and centrifuged at 10,000*g* for 1 min. The Triton X-100/S-EDTA-soluble and -insoluble proteins were collected from the supernatant and pellet, respectively.

### In Vitro Dephosphorylation by Alkaline Phosphatase

CIP (New England Biolabs, Ipswich, MA) can be used to release phosphate groups from phosphorylated tyrosine, serine, and threonine residues (Johnson et al., 1997). Proteins were dephosphorylated according to manufacturer's instructions. Briefly, 1.75–3.5 U of CIP/µg protein was incubated with protease inhibitors at 37°C for 30 min. CIP-treated proteins were then subjected to immunoblotting with an anti-TPI1 (10713-1-AP, rabbit polyclonal antibody; ProteinTech Group, Chicago, IL) or anti-AKAP4 antibody, as described below. The antibody against AKAP4, originally assigned as anti-AKAP82, has been previously characterized (Carrera et al., 1994).

### Protein Extraction and Immunoblot Analysis

The spermatogenic cells and sperm cells were concentrated by centrifugation, washed in 1 ml of PBS, resuspended in sample buffer (58 mM Tris–HCl, pH 6.8, 1.7% SDS, 6% glycerol, 100 mM dithiothreitol [DTT]), and boiled for 5 min. Detergent-extracted proteins were mixed with sample buffer and boiled for 5 min. After centrifugation, the supernatants were recovered and saved; protein concentrations were determined by the BCA Protein Assay Kit-Reducing Agent Compatible (Pierce Chemical Co., Rockford, IL). Other tissues were homogenized, sonicated, and boiled in sample buffer without DTT. After centrifugation, the supernatants were recovered and saved. Protein concentrations were determined by the Micro BCA Protein Assay Kit (Pierce Chemical Co.) and then DTT and bromophenol blue were added to final concentrations of 100 mM and 0.002%, respectively. The samples were boiled for 5 min, and protein samples (10 or 20 µg/lane) were separated by SDS–PAGE in 10% or 12% polyacrylamide gels for AKAP4 or TPI1, respectively (Laemmli, 1970). The gels were then transferred to polyvinylidene difluoride membranes (Towbin et al., 1979). After the membranes were blocked with TBST (125 mM NaCl, 25 mM Tris–HCl, pH 8.0, 0.1% Tween-20) containing 5% nonfat dry milk, they were incubated with primary antibody for 1 hr (1.05 µg/ml anti-TPI1 or 1:10,000 anti-AKAP4). After washing with TBST, the blots were incubated for 1 hr with secondary antibody (1:5,000 alkaline phosphate-conjugated goat anti-rabbit IgG whole antibody [GE Healthcare, Milwaukee, WI] in 5% nonfat dry milk in TBST). After washing again with TBST, the bound enzyme was developed with Enhanced Chemifluorescence Western Blotting Reagent Pack (GE Healthcare) according to the manufacturer's directions. Images were produced using a Storm 860 scanner (GE Healthcare). Negative controls using normal sera or secondary antibody-alone were also used to check for specificity.

### Indirect Immunofluorescence Analysis

One million caput or cauda epididymal sperm, collected as described above, were attached to polylysine-coated coverslips for 15 min, fixed with 4% paraformaldehyde in PBS for 15 min, and then permeabilized with methanol for 2 min at −20°C. Permeabilization with methanol was omitted when preparing S-EDTA-treated cauda epididymal sperm. After extensive washing with PBS, the coverslips were incubated at 4°C overnight with 10% goat serum in PBS (blocking solution). The following day, coverslips were incubated at 37°C for 1 hr with primary antibody (2.1 µg/ml anti-TPI1) diluted in blocking solution. After washing with PBS, the coverslips were incubated for 1 hr at 37°C with Alexa Fluor 488-conjugated goat anti-rabbit IgG (H + L) secondary antibodies (1:500; Life Technologies, Carlsbad, CA) diluted in blocking solution. Finally, the coverslips were mounted on slides using 15 µl of Fluoromount-G (Southern Biotechnology Associates, Birmingham, AL), observed with a Nikon Eclipse TE 2000-U inverted microscope (Nikon Instruments, Melville, NY), and photographed with a CFW-1310C digital FireWire camera (Scion, Frederick, MD) using the NIH ImageJ imaging software available online (http://rsb.info.nih.gov/ij/). Nomarski differential interference contrast micrographs were photographed in parallel with the fluorescence images. Negative controls using normal sera or secondary antibody-alone were used to check for specificity.

### Preparation of RNA

Messenger RNAs were prepared from brain, heart, kidney, testis, and ovary using Dynabeads mRNA Direct Kit (Invitrogen, Grand Island, NY) according to manufacturer's instructions for Northern blot hybridization. Total RNA from mixed germ cells was used for 5′- and 3′-RACE analyses.

### Northern Blot Hybridization

Northern blot hybridization was performed similarly to a previous method (Cao et al., 2011). Messenger RNAs (0.5 µg each) from brain, heart, kidney, testis, and ovary were separated by electrophoresis in a 1% agarose gel, containing formaldehyde, and transferred to Hybond N+ membranes (GE Healthcare). The probes were derived from an initial PCR product (*Tpi1* [accession number: NM_009415] F: 5′-TGACCTTCAGAGACTTGAGCC and R: 5′-CGGTGGGAGCAGTTACTAAAC; product size: 851 bp) and ligated into a pCRII-TOPO vector (Invitrogen). The probe was sequenced, then quantified by comparison to a *Hind* III ladder (New England Biolabs) and labeled with digoxigenin (DIG)-dUTP using in vitro transcription (DIG Northern Starter Kit, Roche). The probe was used for hybridization to Hybond N+ membranes at 68°C for 2 hr. Membranes were washed to a stringency of 1× SSC (diluted from 20× SSC: 3 M sodium chloride, 0.3 M sodium citrate, pH 7.0), then 0.05× SSC and 0.1% SDS up to 68°C. Immunological detection with anti-DIG antibody and chemiluminescence reaction with CDP-Star were used to assess the presence of *Tpi1* RNA following the manufacturer's instructions.

### 5′- and 3′-Rapid Amplification of cDNA Ends, Cloning of Tpi1, and cDNA Sequence Analyses

Mixed germ cell amplicons, produced as described above, were cloned into a pCR2.1-TOPO vector (Invitrogen). Plasmid DNA was prepared and sequenced as directed by the manufacturer's instructions. The initial primer designs were based on the *Mus musculus Tpi1* mRNA sequence (NM_009415). The RLM-RACE kit (Ambion, Austin, TX) was used for 5′- and 3′-RACE, and 1 µg of total RNA aliquots were used as templates for reverse transcription with the supplied 5′- (5′-GCUGAUGGCGAUGAAUGAACACUGCGUUUGCUGGCUUUGAUGAAA) and 3′- (5′-GCGAGCACAGAATTAATACGACTCACTATAGGT-12VN) RACE adapters. The cDNA was then subjected to PCR using the 5′- (5′-GCTCATGGCGATGAATGAACACTG) and 3′- (5′-GCGAGCACAGAATTAATACGACT) RACE primers, which were complementary to the anchored adapter, and a primer specific for *Tpi1* (5′-CCCAAGTTCTACTGGATGTCTG). Gradient PCR was used to optimize annealing temperatures. All PCR products were sequenced in both directions. The cloned sequences were analyzed using MacVector Software v12.0.3 (MacVector, Inc., Cary, NC).

## Abbreviations:

CIP: calf intestinal alkaline phosphatase
DIG: digoxigenin
DTT: dithiothreitol
ECF: enhanced chemifluorescence
PBS: phosphate-buffered saline
RACE: rapid amplification of cDNA ends
TPI1: triosephosphate isomerase 1

## Supporting Information

Figure S1 50 RACE of mouse mRNA. Utilization of 50 RACE identified the 50-UTR sequence of Tpi1 cDNAs from male mixed germ cells. Alignment of the four Tpi1 cDNA sequences: NM_009425.1 containing all three potential initiation codons, NM_009415.2 containing all three potential initiation codons, 50 RACE Transcript 1 containing all three potential initiation codons, and 50 RACE Transcript 2 containing only the second and third potential initiation codons.

Figure S2 30 RACE of mouse mRNA. Utilization of 30 RACE identified the 30-UTR sequence of Tpi1 cDNAs from male mixed germ cell populations. Alignment of the three Tpi1 cDNA sequences: NM_009425.1 is not predicted to contain a germ cell-specific 30-UTR, NM_009415.2 is predicted to contain a germ cell-specific 30-UTR, and the 30 RACE sequence. 30 RACE did not detect a predicted male germline-specific 30-UTR in mouse mixed germ cell cDNAs.

Figure S3 Amino acid sequences of TPI1 isoforms deduced from Tpi1 cDNA. Three putative translation initiation codons in full-length mouse Tpi1 cDNA (NM_009415.1) were discovered. The two orange boxes indicate putative translation initiation codons of the two male germ linespecific TPI1 isozymes, and the green box is the known translation initiation codon of somatic-type TPI1. The italicized numbers above the orange and green boxes indicate the amino acid lengths of the predicted proteins. The red box indicates the in-frame stop codon upstream from the start codons.
