# Supplementary figures and images for: Male Mice Express Spermatogenic Cell-Specific Triosephosphate Isomerase Isozymes
Source: Mol Reprod Dev. 2013 Aug 19;80(10):862–70. doi: 10.1002/mrd.22217 (PMC3916887; doi:10.1002/mrd.22217)

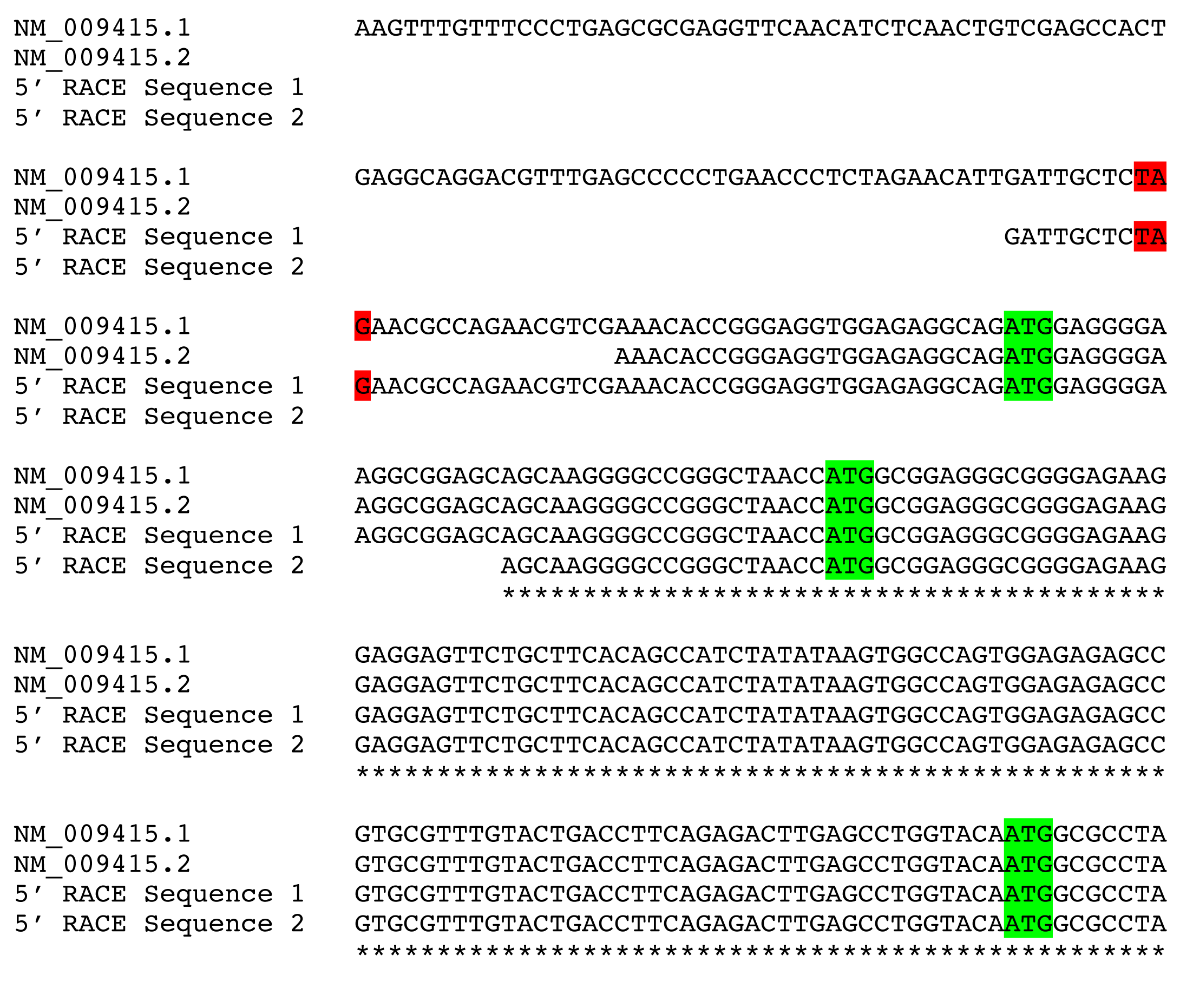

Supplement: Figure S1 — 50 RACE of mouse mRNA. Utilization of 50 RACE identified the 50-UTR sequence of Tpi1 cDNAs from male mixed germ cells. Alignment of the four Tpi1 cDNA sequences: NM_009425.1 containing all three potential initiation codons, NM_009415.2 containing all three potential initiation codons, 50 RACE Transcript 1 containing all three potential initiation codons, and 50 RACE Transcript 2 containing only the second and third potential initiation codons. [file mrd0080-0862-sd1.tif]

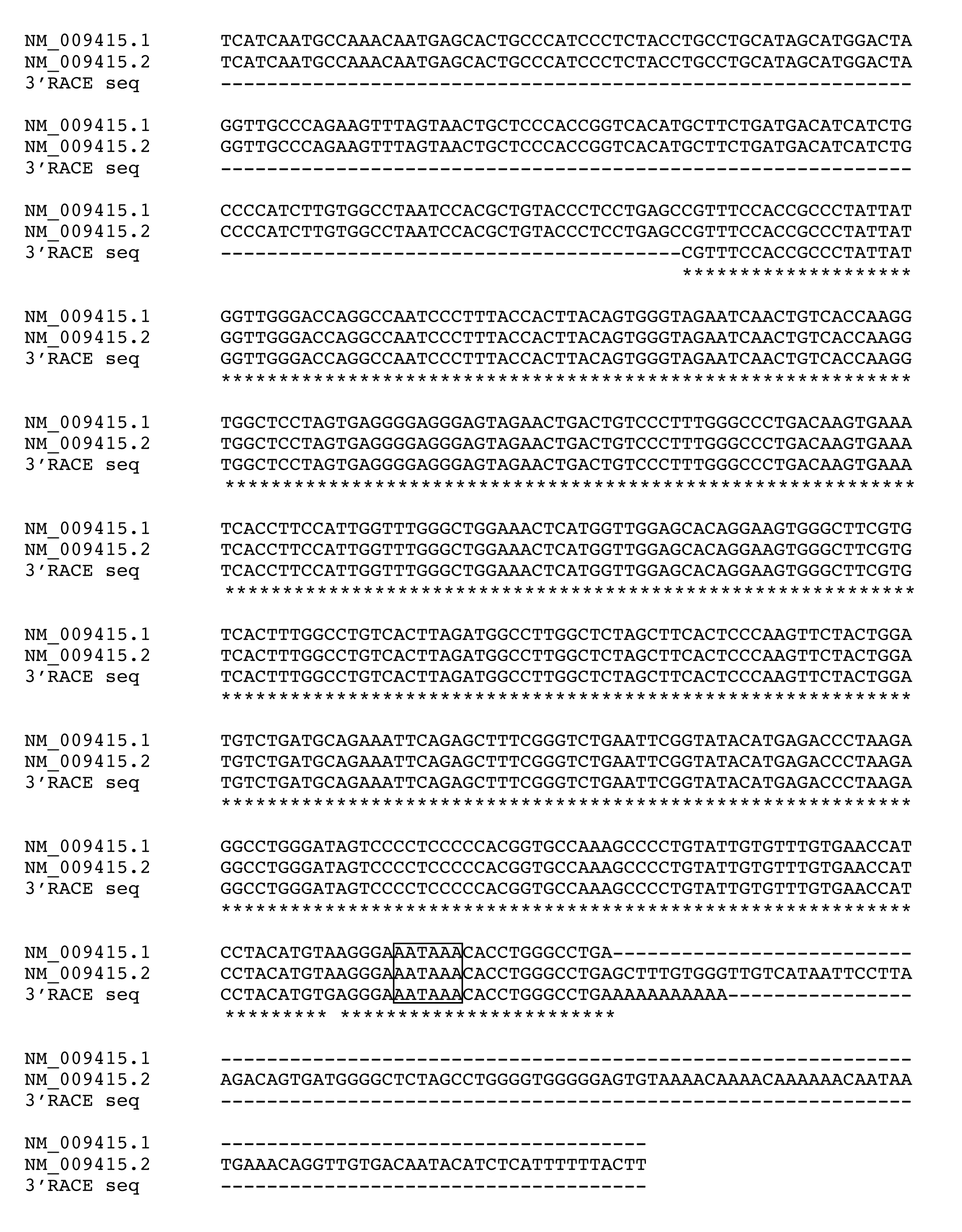

Supplement: Figure S2 — 30 RACE of mouse mRNA. Utilization of 30 RACE identified the 30-UTR sequence of Tpi1 cDNAs from male mixed germ cell populations. Alignment of the three Tpi1 cDNA sequences: NM_009425.1 is not predicted to contain a germ cell-specific 30-UTR, NM_009415.2 is predicted to contain a germ cell-specific 30-UTR, and the 30 RACE sequence. 30 RACE did not detect a predicted male germline-specific 30-UTR in mouse mixed germ cell cDNAs. [file mrd0080-0862-sd2.tif]

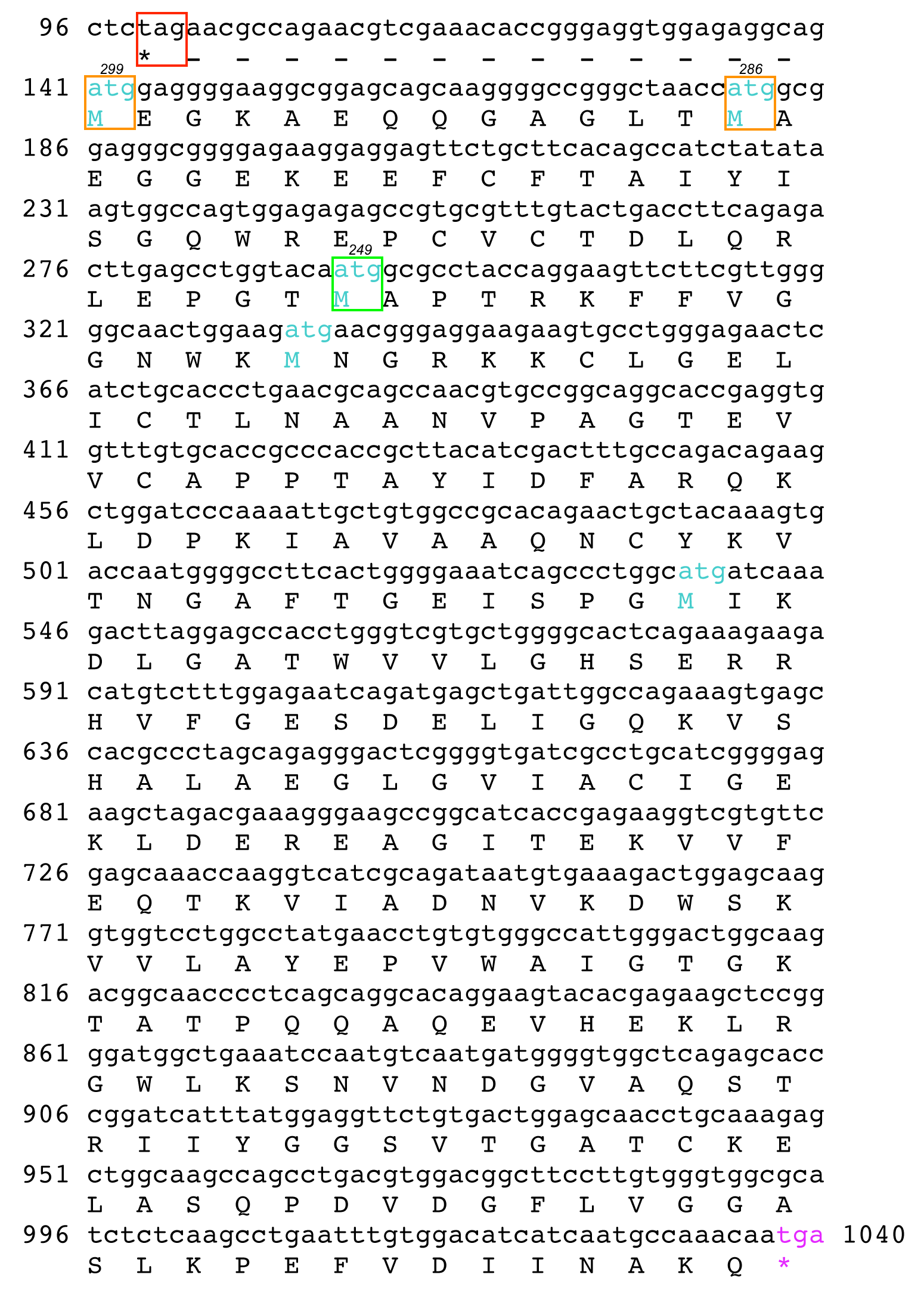

Supplement: Figure S3 — Amino acid sequences of TPI1 isoforms deduced from Tpi1 cDNA. Three putative translation initiation codons in full-length mouse Tpi1 cDNA (NM_009415.1) were discovered. The two orange boxes indicate putative translation initiation codons of the two male germ linespecific TPI1 isozymes, and the green box is the known translation initiation codon of somatic-type TPI1. The italicized numbers above the orange and green boxes indicate the amino acid lengths of the predicted proteins. The red box indicates the in-frame stop codon upstream from the start codons. [file mrd0080-0862-sd3.tif]
